# Polystyrene microplastics induced nephrotoxicity associated with oxidative stress, inflammation, and endoplasmic reticulum stress in juvenile rats

**DOI:** 10.3389/fnut.2022.1059660

**Published:** 2023-01-06

**Authors:** Wanzhen Wang, Jiafu Guan, Yueying Feng, Liju Nie, Yuanyuan Xu, Hengyi Xu, Fen Fu

**Affiliations:** ^1^The Second Affiliated Hospital of Nanchang University, Nanchang, China; ^2^State Key Laboratory of Food Science and Technology, Nanchang University, Nanchang, China; ^3^Jiangxi Maternal and Child Health Hospital, Nanchang, China

**Keywords:** polystyrene microplastics, nephrotoxicity, juvenile, oxidative stress, endoplasmic reticulum stress, inflammation

## Abstract

**Introduction:**

Unintended intake of microplastic particles has been demonstrated to exert adverse health effects, however, studies on relevant nephrotoxicity in juvenile mammals are lacking.

**Methods:**

Therefore, we investigated the potential nephrotoxicity of oral-exposed polystyrene microplastics (PSMPs) (1,000 nm, 2.0 mg/kg/d) for 28 days in juvenile rats. Levels of oxidative stress, inflammation, and endoplasmic reticulum (ER) stress in kidneys were analyzed.

**Results and discussion:**

Results revealed that PSMPs noticeably decreased the growth rate of bodyweight, and organ index of the kidney, cardiac, and ovary. The intestinal injury caused by PSMPs exposure was also observed, which was distinctly alleviated with N-acetyl-cysteine (NAC) and Salubrinal (Sal) treatment compared with the single PSMPs group. PSMPs caused histological lesions of the kidney *via* disrupting the serum blood urea nitrogen (BUN), creatinine (CRE), and pro-inflammatory mediators IL-1β, IL-6, and TNF-α. Furthermore, PSMPs exposure induced ER stress and inflammation presumably potentially mediated by oxidative stress in kidneys of rats. Eventually, PSMPs also promoted renal cells apoptosis, manifested as an obvious increase in the number of positive cells for the dUTP nick end labeling of Terminal deoxynucleotidyl transferase, which also can be confirmed by the elevated expression of genes associated with apoptosis *Bcl-2*, *Bax*, *Caspase-12*, *Caspase-9*, *Caspase-3*, and IHC score of Caspase-12 in the PSMPs group. Supplementation of NAC and Sal not only ameliorated the PSMPs-induced oxidative stress and ER stress but also the inflammation and apoptosis in the kidney. Collectively, this study suggested that PSMPs caused nephrotoxicity in juvenile rats potentially through oxidative damage and ER stress, which call for greater efforts to be taken on regulating the PSMPs ingestion in children.

## 1. Introduction

As the modern lifestyle transfers, the consumption of plastic products has increased. Microplastic (MPs), which has been considered food contamination, is ubiquitous and poses a threat to human health. MPs (1-5,000 μm) are primarily classified into two categories: primary MPs and secondary MPs. The majority of primary MPs are produced during the manufacturing process, in which cosmetic granules, medication transporters, and industrial and technical uses become the most prominent (1). Larger plastics from ordinary products like facial masks, food packaging bags, and clothes, provide the majority of secondary MPs, which enter the environment broken down into tiny pieces via sunlight, ultraviolet radiation, wave energy, thermal decomposition, and additional biological variables (2, 3). According to the study by Geyer et al., approximately 6,300 metric tons of plastic waste has been generated until 2015, with the amount anticipated to rise to about 12,000 metric tons by 2050 (4). MPs can be consumed by marine creatures and have been shown by various studies to be hazardous to the marine ecosystem. With the rising levels of consumption and increasing demands for nutrition, MPs have been a significant issue for food security, food safety, and human health given the increased consumption of fish and other seafood products (5). MPs can serve as a carrier for the adsorption of toxic substances, including heavy metals, organic pollutants, pathogens, and other contaminants. MPs and their co-contaminants can be transferred *via* the food chain and accumulate across different trophic levels, which may have a detrimental effect on human health (6–8). Dietary exposure is one of the main routes for humans exposed to MPs. According to an analysis of the typical annual consumption of MPs from food intake, that number ranges from 39,000 to 52,000 particles (9). Toxic chemicals are consumed by humans mainly through seafood products and drink items, accumulating in the gastrointestinal tract. Despite that increasing evidence demonstrating that MPs contaminate a variety of foods and beverages (9), health risks human-derived from dietary exposure to MPs are rarely assessed. Organisms can digest particles smaller than 150 μm, which may then pass through the intestinal wall and enter lymph nodes and other body organs (10). Recent reports suggested that MPs can accumulate in humans’ lungs and digestive tract and then be scattered into blood, placenta, and female follicular fluid (11–13). In wastewater and surface sediments, polystyrene MPs (PSMPs) are the most often found MPs (14, 15). Chen et al. revealed that PSMPs (3.54 ± 0.39 μm) could be entirely absorbed by human embryonic kidney 293 (HEK293) cells producing cytotoxicity (16). Oral intake PSMPs affect intestinal microbiota and metabolism, diminish intestinal mucosa, and disrupt intestinal barrier function, all of which are detrimental to both human and animal health (17). Deng et al. inferred that PSMPs build up in the liver, kidney, and intestine, causing energy and lipid metabolic disorders along with oxidative stress in mice (18). It is noteworthy that children are more sensitive to chemicals than adults (19). Interestingly, PSMPs are found in a variety of foods and products marketed to children, such as milk, honey, sugar, feeding bottles, and toys (20), promoting children being more vulnerable to MPs than adults. Studies have reported that PSMPs can be transferred through the placenta and generate developmental toxicity (21). Since children’s defensive systems are still developing, studying the impact of PSMPs on their health is critical.

The kidney is vital for the evacuation of fine particulates, particle deposition and buildup in the kidney can cause injury (22). In particular, environmental contaminants, such as air pollution, may increase the risk of chronic kidney disease (CKD) or hasten its course (23). Even though the fact that the kidneys in a juvenile species are far more vulnerable to PSMPs than among an adult, studies on PSMPs-induced nephrotoxicity in juvenile animals are scarce. Oral PSMPs exposure is the main pathway for children. Meng et al. confirmed that PSMPs (4 μm) aggregation is aided through their digestion, which affects the physicochemical characteristics by increasing the Zeta potential value of PSMPs in the digestive process. PSMPs appeared possibly to circulate throughout the body and then collected in the kidneys of mice, causing nephrotoxicity through oxidative stress and inflammatory response (24). Ingested PSMPs could surpass the intestinal barrier and accumulate in organs distant from the gut (25). Inflammatory response and oxidative damage are the primary causative agents of CKD in environmental pollution (23). Besides, oxidative stress, inflammatory harm, and enhanced absorption or translocation are the major effects of microplastic exposure on the body (26). Here, we speculate that PSMPs exposure by oral administration cause nephrotoxicity through oxidative stress and inflammation.

The primary mechanisms of mammalian cytotoxicity caused by PSMPs are oxidative stress and inflammation (27). Numerous investigations on animals show that PSMPs modify intestinal epithelial permeability by inducing oxidative stress and inflammation in the intestinal epithelium (28). PSMPs can also adsorb pollutants and act as transporters of intestinal poisons, resulting in toxicity. The intestinal barrier is crucial in maintaining the equilibrium of the intestinal environment and preventing bacteria and poisons from entering the intestines since it is the first line of defense against external influences (29, 30). Therefore, in the current work, we explored gut barrier damage but not the primary target, but rather the nephrotoxicity induced by PSMPs. Similarly, PSMPs can activate oxidative nephrotoxicity and alter renal barrier integrity *in vivo* and *in vitro* (16, 24). Oxidative stress is the initiator of endoplasmic reticulum (ER) stress, both of which can lead to apoptosis. What’s more, Wang et al. revealed that PSMPs (2 μm) accumulated in mice and human kidney proximal tubular epithelial cells (HK-2 cells), causing excessive reactive oxygen species (ROS) in the mitochondria, and ER stress, inflammatory response, and autophagy (31). The unfolded protein response (UPR) occurs when unfolded or improperly folded proteins build up in the endoplasmic reticulum, which is a specialized location for protein folding and maturation (32). Following the activation of the UPR, ER stress is activated through three primary classical ER stress sensors including inositol-requiring kinase 1 α (IRE1), protein kinase R-like ER kinase (PERK), and activating transcription factor 6 (ATF6) (33). Multiple kidney pathologies, such as CKD, diabetic nephropathy, and renal fibrosis, are linked to ER stress (34, 35). Therefore, we are investigating how PSMPs affect kidney damage in juvenile rats based on oxidative stress, inflammation, and ER stress.

Consequently, our purpose was to explore nephrotoxicity induced by PSMPs exposure with oral administration for 28 days in juvenile rats. Provide a theoretical basis for the mechanism of PSMPs-induced juvenile kidney injury by assessing the levels of oxidative stress, ER stress, inflammation, and apoptosis in kidney tissue.

## 2. Materials and methods

### 2.1. Materials

PSMPs (2.5% w/v, 10 ml) were obtained from Tianjin Baseline ChromTech Research Centre (Tianjin, China). The diameter of 1 μm PSMPs was used in this study. PSMP morphology was recorded by a scanning electron microscope (SEM) (Regulus 8100, Hitachi, Japan). PSMPs were dissolved in deionized water and treated with supersonic wave vibration for 15 minutes to fully suspend before measuring their diameter and zeta potential by the dynamic light scattering (DLS) (Zeta Sizer Nano ZS90, Malvern Instruments Ltd., Britain). Chemical components were analyzed by Fourier transform infrared spectroscopy (FTIR) (Fourier Transform Infrared Spectrometer, Nicolet iS50). N-acetyl-cysteine (NAC) and Salubrinal (Sal) alternately obtained from Beyotime Biotechnology Co., Ltd., and Macklin Reagents, Ltd., in Shanghai.

### 2.2. Animals and treatment

Thirty-two female SD rats (3 weeks old, 40 ± 5 g) were purchased from Nanjing kerys Animal Co., Ltd, (Nanjing, China). Animals were supplied with standard rodent chow and tap water as well as housed adaptively under a standard facility with 24 ± 2°C, a 12 h light and dark cycle for a week accommodation. Animal care and experimentation were approved by the Animal Care Review Committee (approval number, 0064257; Nanchang University, Jiangxi, China). Rats were divided into four groups randomly (*n* = 8), which were the control, the 2.0 mg/kg/d PSMPs group, the NAC group, and the Sal group. The dose used here was decided by an earlier study that claimed PSMPs distinctively cause intestinal damage and intestinal barrier disruption *in vivo* at 2.0 mg/kg (36), which may prompt PSMPs to pass through the intestinal wall to the kidney. In addition, 0.1-0.5 g of MPs may be consumed by humans per week on average in the world, according to research calculations (37). The average rat weighs 100 g, with a daily intake of PSMPs of 0.2 mg and a weekly intake of 1.4 mg, both of which are within the acceptable range for human ingestion. PSMPs were dispersed in deionized water and vibrated by supersonic wave for 15 min before oral gavage. More specifically, PSMPs was diluted with pure water, and rats were administrated with 5 μl/g of the substance daily in the latter three groups. In parallel, an equal quantity of deionized water was given to the control group similarly. In addition, rats from NAC and Sal groups were separately intraperitoneally injected with 100 mg/kg NAC solution and 1.5 mg/kg Sal solution half an hour after the gavage of PSMPs, once every two days, according to previous studies (38, 39). NAC and Sal solution was diluted by sterile saline. The control and PSMPs groups were treated with sterile saline by intraperitoneal injection every two days. After 28 days of exposure, all rats were starved for 12 h and then euthanized under anesthesia on the 29th day. Serum was isolated from blood after centrifugation at 8,000 rpm for 15 min at 4°C. Organs including the colon, kidney, liver, cardiac, ovary, and uterus were collected and wet weight was measured. Serum and tissue storage were kept at −80°C for subsequent examination.

### 2.3. Histological analysis of colon and kidney

The colon of three rats from each group was randomly selected and fixed with 4% paraformaldehyde or Carnoy’s solution. After fixation, the colon tissue was cut into 5 μm thick sections embedded in paraffin. Each colon sample was in both stained with H&E and Alcian blue- Periodic Acid Schiff (AB-PAS) staining for histological analysis. A Nikon T1 optical microscope (Tokyo, Japan) was used for photographing to evaluate the epithelial injury and inflammatory infiltration. The epithelial injury was evaluated by scoring with different indices (0, intact; 1, slight epithelial lesion; local goblet cell loss and mucosal erosion; 3, extensive goblet cell loss and mucosal damage extending to muscular) (40). The inflammation was scored according to the previous study (41). The mucus coverage rate from AB-PAS staining was measured by ImageJ software.

The kidney was fixed in 4% paraformaldehyde for histopathological analysis. After fixation for 24 h, kidney samples were chopped into 5-6 μm thick slices. H&E stain was applied to the slides. The stained sections were fixed on the glass side and observed under a light microscope (Tokyo, Japan).

### 2.4. Biochemical measurement and ELISA analyses

The creatinine (CRE) in serum and blood urea nitrogen (BUN) were assessed using commercial kits (Jiancheng, Nanjing). Inflammatory markers in serum including tumor necrosis factor-α (TNF-α), interleukin1β (IL-1β), and IL-6 levels were measured by ELISA kits (Yansheng, Shanghai). All operations were performed according to the kit’s instructions.

Kits (Jiancheng, Nanjing) were used to determine the oxidative stress indicators glutathione peroxidase (GSH-PX), superoxide dismutase (SOD), and malondialdehyde (MDA) in renal tissue. Using a Bradford protein concentration test kit (Applygen Technologies Inc., Beijing, China), the protein content of tissue homogenates was evaluated. Each experiment was carried out precisely following the kit’s instructions.

### 2.5. RT-qPCR analysis

Total RNA was extracted from kidney and colon tissue using MolPure^®^ TRIeasy Plus Total RNA Kits (Yepsen Biotech Co., Ltd., Shanghai). Using a NanoDrop (ND-100) Spectrophotometer (Thermo scientific Inc., USA), the purity and quantity of total RNA were evaluated. After being adjusted to the same concentration, reverse transcription was performed with Hifair^®^ III 1st Strand cDNA Synthesis SuperMix for qPCR (gDNA digester plus) (Yepsen Biotech Co., Ltd., China). The primers were synthesized by General Biosystems Co., Ltd, (Anhui, China) and validated successfully. What’s more, Agilent’s AriaMx Real-Time PCR application (Agilent Technologies, USA) was used to conduct real-time quantitative PCR (RT-qPCR). Supplementary Table 1 contains the primer information.

### 2.6. Immunohistochemistry (IHC) analysis

Partial paraffin sections of kidney tissue were dewaxed with xylene and absolute ethanol with different concentration gradients. For antigen repair, tissue slices were put in a microwave oven containing citric acid (pH = 6.0, G1202) antigen retrieval solution. After natural cooling, slides were placed in PBS (pH = 7.4) and washed three times with shaking on a decoloring shaker, for 5 min each time. To block endogenous peroxidase, sections were placed in a 3% hydrogen peroxide solution (Service, G0115) and incubated at room temperature for 25 min in the dark. Following the incubation at room temperature for 30 min with sealing solution (3% Bovine Serum Albumin), sections were added the primary antibody GRP78 (1:200, GB11098), NF-κB/p65 (1:200, bs-0465R) and Caspase-12 (1:100, GB111695) prepared in a proportion and incubated at 4°C overnight. The secondary antibody- horseradish peroxidase (HRP) (1:200, G23303) labeled goat anti-rabbit was dripped on slices, and slices were added diaminobenzidine (DAB) chromogenic solution to develop color. Finally, brown-yellow is a positive expression, and ImageJ software was used for immunohistochemical quantification.

### 2.7. Terminal deoxynucleotidyl transferase dUTP nick end labeling (TUNEL) staining assay

The TUNEL staining procedure was as follows. Briefly, the kidney tissue wax block was dewaxed with xylene and then hydrated with gradient absolute ethanol. Circles were drawn around the tissue with a histochemical pen once the sections dried. Then, the tissue was covered with proteinase K working solution dropwise. After 22 min at 37°C, PBS was used to decolorize the tissue. Following the drying of the sections, 0.1% Triton X-100 solution was poured dropwise in a circle to cover the tissue. The tissue was then incubated for 20 min at room temperature and rinsed three times with PBS. Reaction solution was added to the sections according to the instructions of the CF488 TUNEL Cell Apoptosis Detection Kit (Servicebio, G1501). Slices were incubated with DAPI solution at room temperature for 10 min to counterstain the nucleus. DAPI labeling turned the nucleus blue. Green cells represent positive apoptosis. DAPI emits blue light with a wavelength of 420 nm, and FITC emits green light with an excitation wavelength of 465–495 nm and an emission wavelength of 515–555 nm. Images were collected through a fluorescence microscope. The number of apoptotic cells was counted under the ImageJ software. Five non-overlapping fields of view were randomly selected in each slide to count the number of apoptotic cells.

### 2.8. Statistical analysis

One-way analysis of variance (ANOVA) and Tukey’s multiple comparisons test was used to compare the results between multiple groups. SPSS 26.0 was used to determine the significance of the difference between the groups. GraphPad Prism 8.0.1 was used to create the statistical diagrams, which show all data as mean SD. The differences were deemed significant at *p* < 0.05.

## 3. Results

### 3.1. Characterization of PSMPs

**FIGURE 1:**
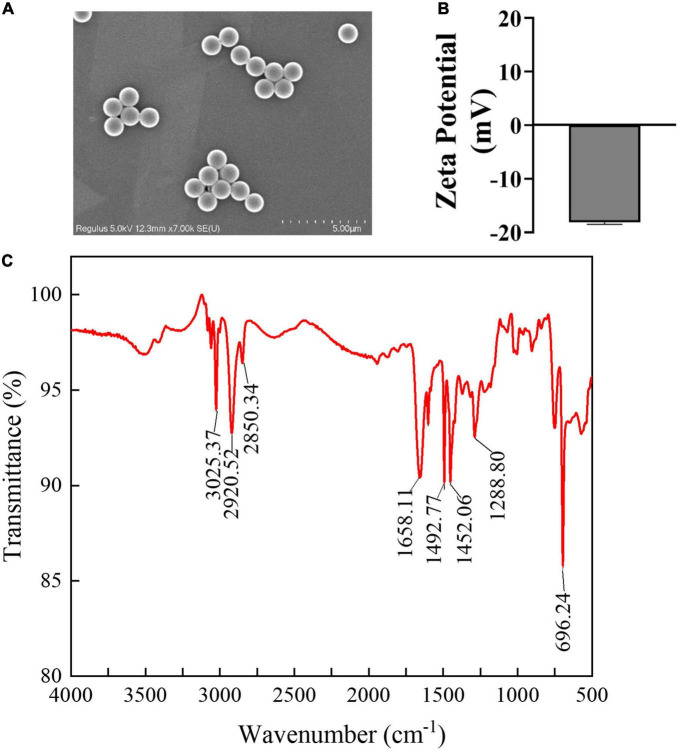
Characterization of PSMPs. **(A)** Scanning electronic microscopy was used to measure the morphology and properties of PSMPs. **(B)** Zeta potential of PSMPs in deionized water was examined by DLS. **(C)** FTIR image of PSMPs.

Scanning electron microscope analyses of PSMPs size and shape revealed that all particles have a smooth surface, spherical morphology, and a uniform size of 1,000 nm (Figure 1A). Furthermore, Figure 1B shows that the zeta potential of PSMPs is low (approximately –18.4 mV). The FTIR result (Figure 1C) indicates that the major constituent of PSMPs is polystyrene (16, 42).

### 3.2. Alterations of biological changes after PSMPs exposure

**FIGURE 2:**
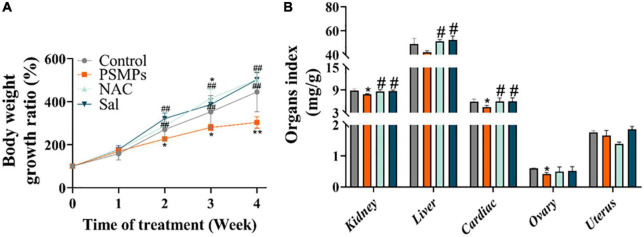
Alterations of biological changes induced by PSMPs. **(A)** Bodyweight growth ratio. **(B)** Organ index. *p < 0.05, **p < 0.01, compared with the control. #*p* < 0.05, ##*p* < 0.01 compared with the PSMPs group. Data are presented as mean ± SD.

Body weight was measured once a week. The weight gain in the 2.0 mg/kg PSMPs group significantly decreased by about 43.14% from the second week compared with the control group (*p* < 0.05). Rats undergoing NAC and Sal treatments began to gain more body weight than those in the PSMPs group by the second week. The body weight growth ratio of the NAC and Sal groups was 59.96% (*p* < 0.01) and 94.23% (*p* < 0.01) higher than th in the PSMPs group (Figure 2A), separately. In addition, the organ indexes of rats, including the kidney, liver, cardiac, ovary, and uterus, were measured (Figure 2B). The renal and cardiac organ coefficients in the PSMPs group dramatically fell by 11.4 and 23.7%, respectfully, in comparison to the control group. But the two organ coefficients in the NAC and Sal group noticeably rose by 9.0 and 10.2% as compared to the PSMPs group. However, no notable alterations in the liver organ index were found between the 2.0 mg/kg PSMPs group and the control (*p* = 0.062). NAC and Sal may raise the liver organ coefficient but not the ovarian organ coefficient. The uterine organ index was hardly altered among the four groups.

### 3.3. Assessment of the colon epithelial injury and mucus secretion after oral exposure to PSMPs for 28 days

**FIGURE 3:**
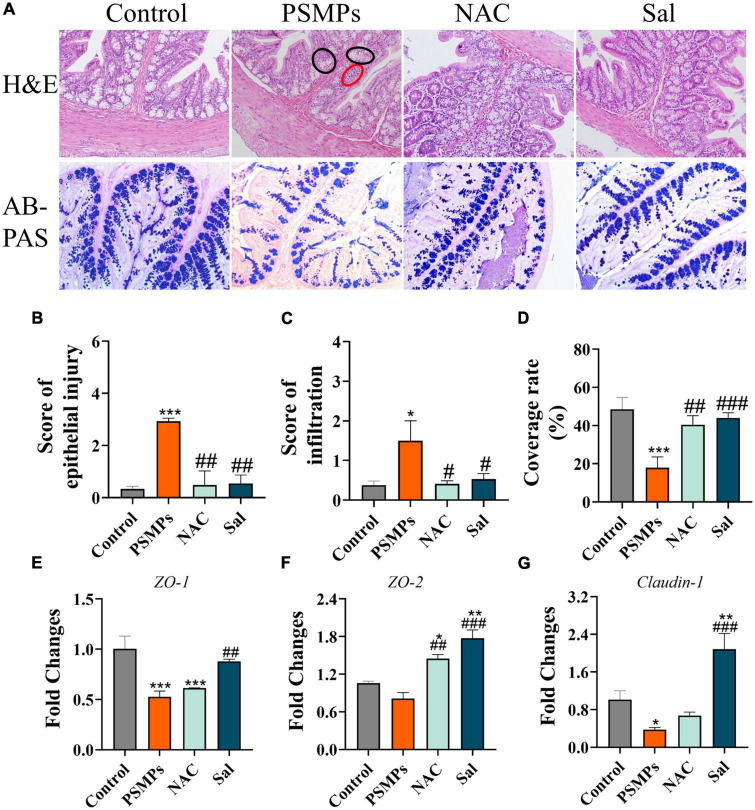
Evaluation of the colon epithelial injury and mucus secretion after oral exposure to PSMPs in juvenile rats. **(A)** Representative images of H&E staining (magnification 100×), red circle: infiltration of inflammatory cell, black circle: crypt shrinkage and structural disorder. Typical micrographs of AB-PAS staining (magnification 100×). **(B,C)** The score of epithelial injury and inflammation. **(D)** The coverage rate of mucus. **(E–G)** Relative expression of genes *ZO-1*, *ZO-2*, and *Claudin-1* in the colon. *p < 0.05, **p < 0.01, ***p < 0.001, compared with the control. #*p* < 0.05, ##*p* < 0.01, ###*p* < 0.001 compared with the PSMPs group. Data are presented as mean ± SD.

H&E and AB-PAS staining were used to evaluate intestinal barrier disruption, epithelial damage, and inflammatory infiltration in the colon. Compared with the control, the histological results of the colon from H&E staining showed that PSMPs exposure at a dose of 2.0 mg/kg per day caused local colon injury, including inflammatory cell infiltration, crypt shrinkage, and structural disorder (Figure 3A). Epithelial injury and inflammatory infiltration scores in the PSMPs group were considerably higher than those in the control group and consistent with the histopathologic findings. Additionally, the PSMP group’s colonic AB-PAS staining results revealed a reduction in goblet cell count and mucus secretion (Figures 3A, D), which indicated that oral PSMPs exposure injured the colon and disrupted the intestinal barrier. However, NAC and Sal effectively ameliorated the colonic injury and facilitated mucus secretion (Figures 3B–D) compared with the PSMPs group. The transcription levels of genes in the colon related to the tight junction (ZO-1, ZO-2, and Claudin-1) exhibited the same trend (Figures 3E, F). The results suggested that the intestinal barrier was negatively influenced by PSMPs exposure, and NAC and Sal may relieve the injuries caused by PSMPs.

### 3.4. Histological changes in kidney and alterations of serum indicators representing the renal function

**FIGURE 4:**
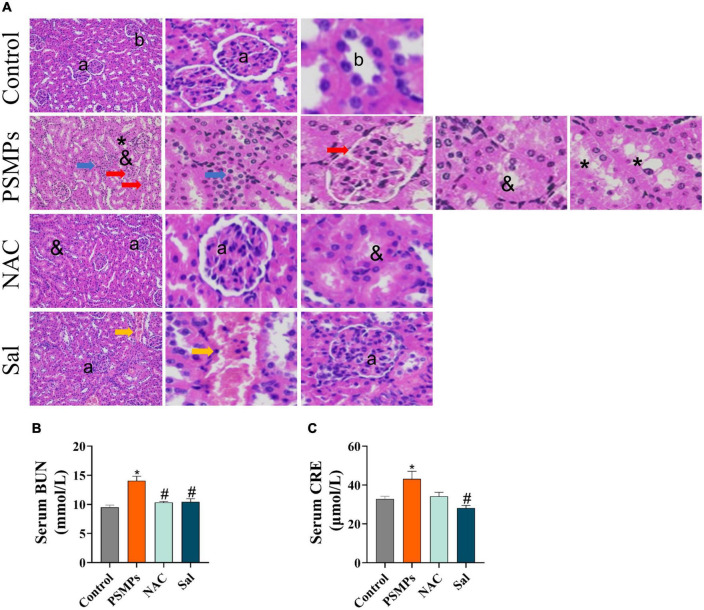
Changes of pathophysiology in the kidney and serum biochemical indicators of renal function induced by PSMPs exposure. **(A)** Representative images of rat kidneys with H&E staining (magnification 200×). a: normal glomerular structure; b: normal tubular structure. blue arrow: inflammatory cells infiltrate in the renal interstitium; red arrow: the glomerular division, yellow arrow: the renal interstitial hemorrhage; &: absence of brush border in proximal tubule epithelial cells; asterisk: detachment of distal tubular epithelial cell. Scale bars = 100 μm. **(B)** The level of serum BUN. **(C)** The level of serum CRE. *p < 0.05, compared with the control. #*p* < 0.05 compared with the PSMPs group. Data are presented as mean ± SD.

The kidney structure of rats was analyzed according to the previous study (24) and the normal glomerular and tubular structures were represented by the letters a and b in the control (Figure 4A). PSMPs exposure induced obvious pathological damage to kidney tissue, including the glomerular division (red arrow), inflammatory infiltration (blue arrow), missing brush border (&), and detachment of renal tubular epithelial cells (asterisk). In NAC and Sal groups, the kidney structure was close to the control group while the renal interstitial hemorrhage was detected in the Sal group (yellow arrow). As for the CRE and BUN levels in serum which could represent renal function, PSMPs exposure markedly increased levels of serum CRE (43.18 ± 3.93 μmol/L) and BUN (14.27 ± 0.78 mmol/L) than the Control group (32.91 ± 1.30 μmol/L for CRE, 9.50 ± 1.38 mmol/L for BUN). With the NAC and Sal treatment, the level of BUN (10.32 ± 0.20 mmol/L, 10.43 ± 0.53 mmol/L) was lower than the PSMPs group (*p* < 0.05). The level of CRE showed a downtrend in the NAC group (34.24 ± 2.01 μmol/L) (*p* = 0.06) and a substantial decrease in the Sal group (28.18 ± 1.37 μmol/L) (*p* < 0.05) compared with the PSMPs group.

### 3.5. Oxidative stress indicators

**FIGURE 5:**
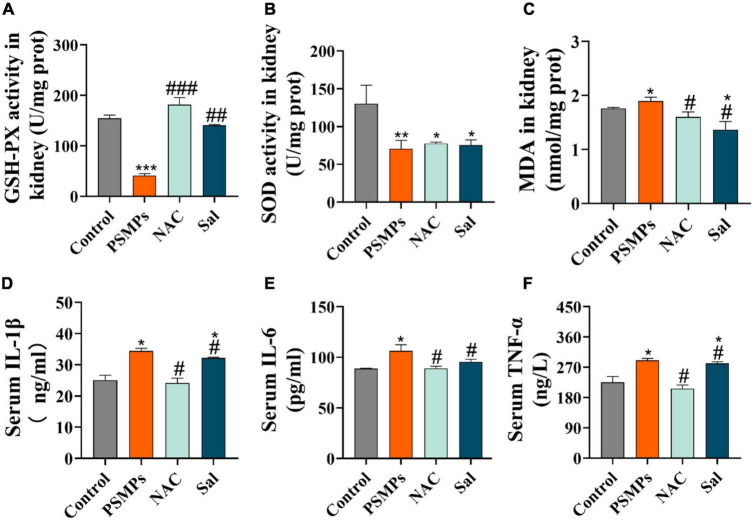
Systematic inflammatory response and renal oxidative stress induced by PSMPs exposure. **(A)** GSH-PX, **(B)** SOD, **(C)** MDA, **(D)** IL-1β, **(E)** IL-6, and **(F)** TNF-α. *p < 0.05, **p < 0.01, ***p < 0.001, compared with the control. #*p* < 0.05, ##*p* < 0.01, ###*p* < 0.001, compared with the PSMPs group. Data are presented as mean ± SD.

GSH-PX, SOD, and MDA were measured in kidney tissue as indicators of oxidative stress. The levels of antioxidant enzymes GSH-PX and SOD were particularly decreased in the PSMPs group (40.92 ± 3.99 U/mg for GSH-PX, 70.40 ± 11.30 U/mg for SOD) compared with the control group (154.52 ± 6.67 U/mg for GSH-PX, 130.34 ± 24.41 U/mg for SOD). GSH-PX concentrations in kidneys were 2.5 times higher in the Sal group and 3.5 times higher in the NAC group when compared to the PSMPs group (Figure 5A), however, there were almost no changes in the activity of SOD (Figure 5B). In the PSMPs group, lipid peroxidation index MDA levels were 0.14 nmol/mg higher than in the control group (*p* < 0.05). The level of MDA was dramatically reduced with the NAC and Sal intervention compared to the PSMPs group (Figure 5C).

### 3.6. Assessment of serum inflammation levels

Three inflammatory markers which are referred to as TNF-α, IL-6, and IL-1β in serum were examined by their corresponding ELISA kits. In this work, we discovered that orally ingested PSMPs causes systemic inflammation, as evidenced by increased levels of three pro-inflammatory cytokines in the serum. Among them, TNFα increased by 28.8%, IL-6 increased by 19.70%,and IL-1β grew by 37.32% compared with the control group. However, following NAC and Sal treatment, the inflammatory response was alleviated by the reduction of these cytokines (Figures 5D–F).

### 3.7. Analysis of the mechanism of kidney injury at the gene level

**FIGURE 6:**
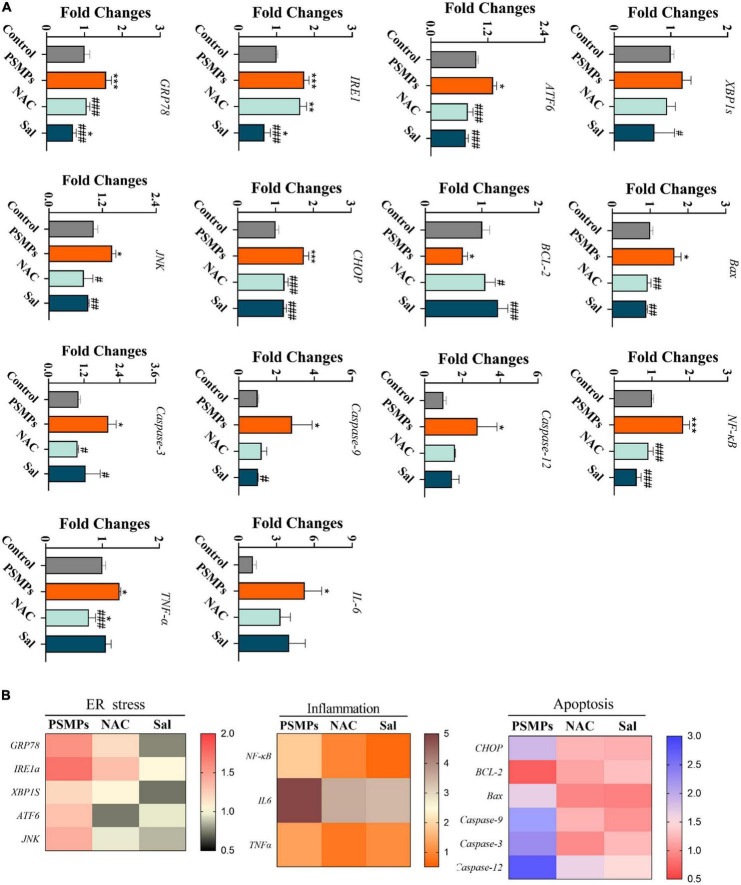
Relative expression of genes associated with ER stress, inflammation, and apoptosis in the kidney after PSMPs oral exposure. **(A)** Relative expression of genes. **(B)** Heat maps of mRNA expression changes. *p < 0.05, **p < 0.01, ***p < 0.001, compared with the control. #*p* < 0.05, ##*p* < 0.01, ###*p* < 0.001, compared with the PSMPs group. Data are presented as mean ± SD.

As is shown in Figure 6A, we deeply analyzed the mechanism of renal injury induced by PSMPs exposure at the gene level, including ER stress-related genes (*GRP78* (78-kD glucose-regulated protein), *IRE1*, *XBP1s* (X-box binding protein 1 splicing), *ATF6*, *JNK* (c-Jun N-terminal kinase) and *CHOP* (the transcription of CCAAT/enhancer-binding protein (C/EBP) homologous protein)), inflammation-related genes (*NF*-κ*B* (nuclear factor kappa B), *TNF*-α and *IL-6*) and genes connected to apoptosis (*Bax*, *Bcl-2*, *Caspase-3*, *Caspase-9*, and *Caspase-12*). Eventually, the relative mRNA expression levels of all the genes exhibited the same pattern, except that of the anti-apoptotic gene Bcl-2, which displayed the opposite pattern. Relative mRNA expression levels of genes were considerably increased in the 2.0 mg/kg PSMPs group compared to the control group and downregulated upon NAC and Sal intervention. Three heat maps showed the relative expression of genes linked to ER stress, inflammation, and apoptosis (Figure 6B).

### 3.8. Immunohistochemical analysis and apoptotic cells analysis

**FIGURE 7:**
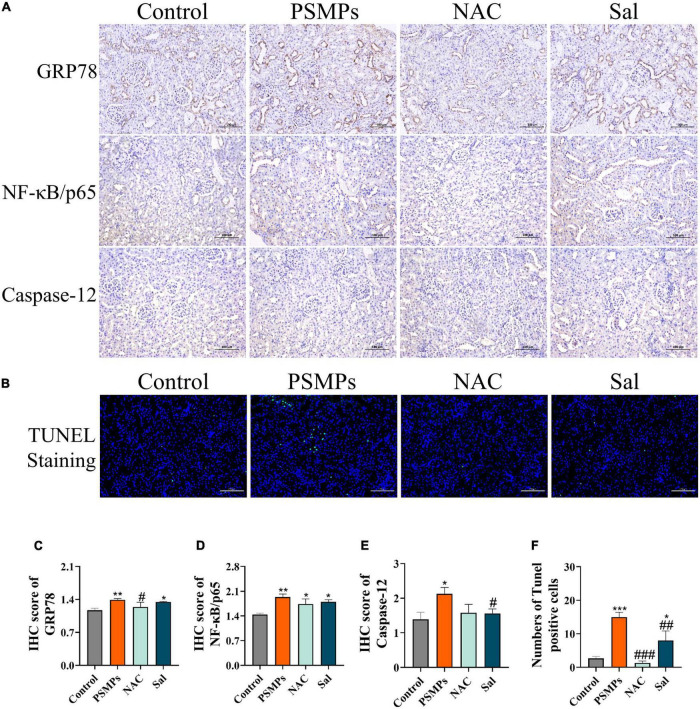
Inflammation and apoptosis in kidney cells induced by PSMPs exposure. **(A)** Representative images of GRP78, NF-κB/p65 and Caspase-12 rat kidney immunohistochemistry (IHC) staining (brown for the positive part) and IHC score of GRP78, NF-κB/p65 and Caspase-12 **(C–E)**. **(B,F)** TUNEL staining (Green fluorescence represents apoptotic cell nuclei and blue fluorescence represents non-apoptotic cell nuclei) and numbers of Tunel-positive cells. Scale bar = 100 μm. *p < 0.05, **p < 0.01, ***p < 0.001, compared with the control. #*p* < 0.05, ##*p* < 0.01, ###*p* < 0.001, compared with the PSMPs group. Data are presented as mean ± SD.

In line with the IHC results, protein levels of Caspase-12, NF-κB/p65, and GRP78 were all vastly greater in the PSMPs group as compared to the control group. Both proteins’ levels dropped compared with the PSMPs group after the two blockers were added (Figures 7A, C, D). The numbers of TUNEL-positive cells were substantially greater in the PSMPs group than in the control group, yet dramatically decrease in the NAC and Sal groups than in the PSMPs group (Figures 7B, E).

## 4. Discussion

Accidental intake of MPs particles in the diet may have adverse health effects. The nephrotoxicity of MPs has been fully elucidated, especially for mammals in the early stage of development. In the present study, PSMPs were administered to weaning rats for 4 weeks to explore the nephrotoxicity and latent mechanism focusing on oxidative stress and ER stress. Moreover, the antioxidant NAC and ER stress blocker Sal was used to solidly verify the mechanism lying in the PSMPs-induced nephrotoxicity.

The particle size of MPs has been demonstrated to be crucial for their biological toxicity in previous studies (43). As biotoxicity increases with decreasing particle size, nanoplastics are potentially more hazardous to living things than microplastics because they are more flexible and reactive to access remote sites and enter cells (44). Treated with PSMPs (2 μm, 0-0.8 mg/ml) induced an obvious accumulation in kidney cells *in vitro*, and PSMPs (0.4 mg/d PSMPs for 8 weeks) were also found to accumulate in kidneys of mice *in vivo*, causing mitochondrial malfunction, ER stress, inflammation, and autophagy (31). In this study, PSMPs were characterized as nearly spherical particles with negative potential and a size of 1.0 μm (Figure 1). And Meng et al discovered that 4 μm fluorescent PSMPs can pass the intestinal barrier and accumulate in mouse kidney tissue (5 mg, gavage after 24 h) using transmission electron microscopy and fluorescence microscopy (24). In this study, we supposed that PSMPs with a diameter of 1.0 μm penetrate the intestinal barrier and accumulate in the kidney, triggering a series of reactions in juvenile rats.

Microplastic possibly affect growth by inducing undesirable satiety by inflaming the gastrointestinal tract, altering the intestinal barrier, and decreasing mucus secretion (45, 46). In the present study, oral exposure to PSMPs elicited a marked decline (almost by 43.14%) in the body weight growth ratio compared to the control group from the second week, which was probably due to the intestinal injury supported by our results (Figures 2A, 3). NAC and Sal have been demonstrated to protect against the harmful effects of nano-ZnO on mammal growth and development (180 mg/kg/d ZnO Nanoparticles for 18 days) (38). Correspondingly, NAC and Sal also alleviated the growth block induced by PSMPs. Additionally, compared to the control group, the renal, cardiac, and ovarian organ coefficients were 11.4, 23.7, and 30.0% lower than that of PSMPs-exposed group, respectively (Figure 2B). In disagreement with our results, Liu et al. found that there were no obvious changes of organ indexes, including the cardiac, liver, kidney, and ovary in mice after PSMPs (30 mg/kg/d) exposure for 35 days (47). It further indicated that the degree of damage varies with different particle size, concentration, exposure period.

Previous studies have demonstrated the translocation of orally ingested PSMPs into the systemic circulation, and subsequent accumulation in the kidney and other organs, such as the cardiac, lung, and brain (24, 48). In our study, PSMPs exposure caused intestinal inflammation and decreased mucus secretion, which suggested the destruction of the intestinal barrier (Figure 3). Recent evidence showed that human exogenous particles with a diameter of smaller than 150 μm can pass through the gastrointestinal epithelium, while micro- and nanoparticles with a smaller than 2.5 μm are absorbed by cells in Payer’s patches (49). And given the higher bioavailability of MPs particles with small sizes, we anticipated that herein the PSMPs with 1 μm may penetrate the epithelial barrier and accumulate in kidney of juvenile rats (50). The particle size and surface charge altered when PSMPs (50 nm, 300 nm, 600 nm, and 4 μm) were passing through the gastrointestinal tract, consequently eliciting the renal toxicity potentially by triggering oxidative stress and inflammation (exposed 25 mg/ml PSMPs for 4 weeks) (24). In this study, we also observed the lesions of glomerular and renal tubules, characterized by the accumulation of inflammatory cells in the renal interstitium based on histopathological analysis of kidney in the PSMPs group (Figure 4A). Besides, levels of serum CRE and BUN in the PSMPs group were separately 32.21% and 50.21% higher than that of the control group, which is one of the reliable predictors of renal dysfunction in clinical (Figures 4B, C). In line with us, Wang et al. also found the kidney dysfunction of higher urine protein (*p* < 0.001) in PSMPs-treated mice (dosages of 0.2 mg/d and 0.4 mg/d), despite lower CRE serum indices, were detected compared to the control group (31). Conversely, the CRE serum indices were 7.14 and 18.42% lower in the 0.2 mg/d and 0.4 mg/d PSMPs group (8 weeks) than in the sham group due to muscular dysfunction in mice. However, the urine protein level of mice was significantly higher than that of the control group (*p* < 0.001), which indicated CKD (31). Our results also indicated the mitigation of NAC and Sal in kidney toxicity (Figures 4B, C). Given the evidences for critical roles of oxidative stress and ER stress played in multiple acute and chronic renal injuries, we concluded that oral exposure to PSMPs over 28 days induced kidney damage in juvenile rats potentially *via* activating oxidative stress and ER stress.

Oxidative stress and inflammation are typical mechanisms of mammalian cytotoxicity (27). The present study showed that the antioxidant proteins SOD and GSH-PX were significantly down-regulated with PSMPs treatment, while the level of MDA was elevated when compared to the control group (Figures 5A–C). Concurrently, the antioxidant enzymes SOD, CAT, and GSH-PX are well-known for their effective defense against oxidative kidney injury by rebalancing oxidative stress (51). In previous study, exposure of 5 μm PSMPs (10 mg/L) combined with cadmium ions (50 mg/L) for 90 days also triggered the oxidative stress with diminished content of SOD (*p* < 0.01), GSH (*p* < 0.05), CAT (*p* < 0.05) and elevated level of MDA (*p* < 0.01) in mice kidneys (52). Oxidative stress caused by over-produced reactive oxygen species (ROS) disrupts cellular homeostasis and promotes inflammation in most cases (53). Evidence suggested that invaded MPs interacted with immune cells, served as gatekeepers eliminating the toxins, to promote the generation of inflammatory factors (27). Therefore, we determined the levels of pro-inflammatory cytokines in serum (TNF-α, IL-6, and IL-1β) combined with relative expressions of genes *TNF*-α and *IL-6* in kidneys, which indicated that PSMPs contributed to both local and systemic inflammation in juvenile rats (Figures 5D–F). These pro-inflammatory cytokines partially derived from the transcription of NF-κB (54) in kidneys of PSMPs-treated group, which was also confirmed by remarkably increased (*p* < 0.01) gene expression level and IHC score in this study (Figure 7A). In accordance with us, PSMPs also aroused significantly elevated levels of cytokines TNF-α, IL-6, and MCP-1 (*p* < 0.05) in mice kidney, resulting in kidney injury by eliciting oxidative stress and inflammation (27). Notably, the levels of the pro-inflammatory cytokines TNF-α, IL-6, and IL-1β reduced by 28.95, 16.36, and 29.70%, respectively, in the NAC group while decreasing by 3.06, 10.11, and 6.68% in the Sal group (Figures 5D–F), solidly demonstrating the involvement of oxidative stress in PSMPs-induced kidney toxicity in juvenile rats.

**FIGURE 8:**
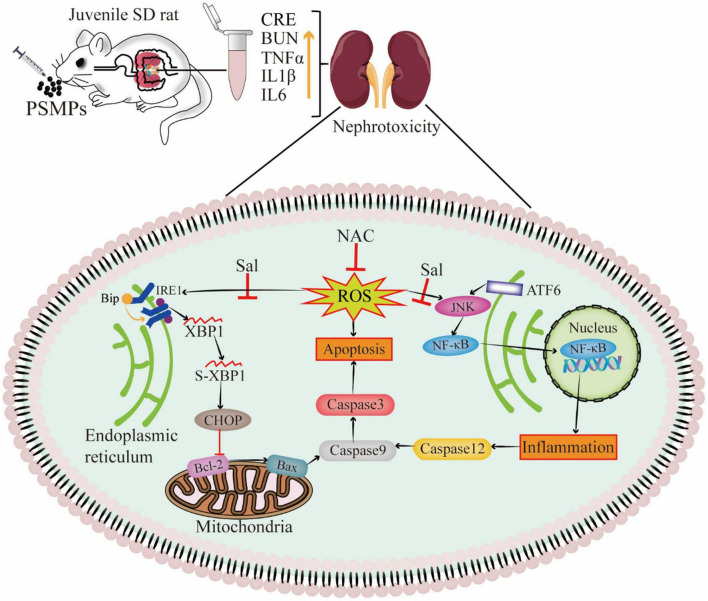
Schematic of mechanism of nephrotoxicity induced by PSMPs oral exposure. (“⊥” inactivation, “↑” increase).

MPs destabilize the nuclear envelope, generate various cell stress, alter damage-associated molecular patterns, and lead to inflammation and apoptosis/necrosis in mammalian cells (27). Another type of cellular stress is ER stress, which has strong links to several acute and chronic renal illnesses (55). The ER lumen contains the GRP78 protein, a significant initiation factor of ER stress. It is well-established that ATF6 and IRE1α control the UPR target gene GRP78 (56). IRE1 changes unspliced XBP1 mRNA into a spliced form that further active XBP1 product, once misfolded proteins accumulate in the ER (57). In line with IRE1, ATF6 dissociates from GRP78 and then moves into the Golgi apparatus. Of note, mRNA levels of *GRP78*, *IRE1*, and *ATF6* were found to be considerably higher in the PSMPs group (Figure 6). The protein level of GRP78 assessed by IHC was increased induced by PSMPs (Figure 7A). Protein misfolding in the ER can be caused by ROS generated by PSMPs either directly or indirectly affecting ER homeostasis (58). JNK is an intermediate substance between oxidative stress and ER stress (59). JNK signaling activation is seen in innate glomerular and tubular cells, as well as invading leukocytes, in most renal diseases (60). In addition, JNK can stimulate NF-κB by boosting the degradation of I-kappaB alpha (IκBα), promoting the inflammatory response, which also well corresponds to the results of inflammation analysis in our study (Figures 6A, 7A) (61). ER stress-mediated activation of Caspase-12 serves a significant role in the apoptosis of proximal tubular cells due to its direct cleavage of Caspase-9 precursors independent of the intrinsic (mitochondrial) pathway to activate Caspase-3 (62). CHOP initiated apoptosis by inhibiting the Bcl-2 functions and promoting the pro-apoptotic Bax (63). By generating pores in the outer mitochondrial membrane, the pro-apoptotic protein Bax causes the efflux of cytochrome c from mitochondria into the cytosol, which activates Caspase-9 *via* apoptotic protease activating factor-1 (Apaf-1) and pro-Caspase-9, followed by Caspase -3 activation (63). Intriguingly, we found that the PSMPs treatment prominently elevated the relative expression of genes related to apoptosis, like *CHOP*, *Bcl-2*, *Bax*, *Caspase-12*, *Caspase-9*, *Caspase-3*, and inflammation, like *JNK*, *NF*-κ*B*, *TNF*-α, *IL-6* compared with the control group (Figure 6), which is consistent with the prior study (31). The effects of PSMPs on apoptosis and inflammation in kidneys were recovered when oxidative and ER stresses were inhibited (Figure 7), suggesting that these effects are probably mediated *via* oxidative stress and the GRP78-IRE1-XBP1s and ATF6-JNK-NF-κB signaling pathways, which were fully illustrated in Figure 8.

Renal damage is observed in juvenile rats exposed to PSMPs, which may lead to chronic kidney disease (CKD), finally evolving into end-stage uremia. It can be demonstrated that the mechanism of PSMPs-induced nephrotoxicity is associated with oxidative stress, inflammation, and ER stress, but there is still no recognized mechanistic pathway. The oxidative stress inhibitor NAC and the ER stress inhibitor Sal dramatically reduced PSMPs exposure-induced nephrotoxicity in rats by inhibiting oxidative stress, inflammation, and endoplasmic reticulum stress signaling pathways. More research should be performed to clarify the internal correlation between that PSMPs exposure caused the intestinal damage and renal toxicity. The number of rats and dosage spectrum hamper the interpretation of our findings. We also need to determine whether PSMPs have dose-dependent effects and whether they target certain organs.

## 5. Conclusion

In brief, exposure to PSMPs caused renal apoptosis and inflammation in juvenile rats associated with the activation of oxidative stress and ER stress. Mechanistically, we found that NAC and Sal protect against nephrotoxicity through the inhibition of oxidative stress and ER stress induced by PSMPs in juvenile rats. As MPs are an emerging food contaminant, the effects on human health risks should be evaluated. Moreover, scholars and governments must take effective measures to safeguard future generations from the omnipresent microplastics.

## Data availability statement

The original contributions presented in this study are included in the article/Supplementary material, further inquiries can be directed to the corresponding authors.

## Ethics statement

The animal study was reviewed and approved by animal care and experimentation were approved by the Animal Care Review Committee (approval number: 0064257; Nanchang University, Jiangxi, China).

## Author contributions

WW and JG: conceptualization, methodology, investigation, validation, and writing – original draft. YF: methodology, formal analysis, and visualization. LN and YX: resources and methodology. HX: writing – review and editing, supervision, and funding acquisition. FF: project administration, supervision, and data curation. All authors revised and approved the final version of the manuscript.
