# Supplementary material for: Polystyrene microplastics induced nephrotoxicity associated with oxidative stress, inflammation, and endoplasmic reticulum stress in juvenile rats
Source: Front Nutr. 2023 Jan 6;9:1059660. doi: 10.3389/fnut.2022.1059660 (PMC9853403; doi:10.3389/fnut.2022.1059660)
Supplement: Supplementary file 1 [file Table_1.docx]

Supplementary Material

Polystyrene microplastics induced nephrotoxicity associated with oxidative stress, inflammation, and endoplasmic reticulum stress in juvenile rats

**Wanzhen Wang, Jiafu Guan, Yueying Feng, Liju Nie, Yuanyuan Xu, Hengyi Xu, Fen Fu**

Supplementary Table 1. Primers used for a quantitative real-time polymerase chain reaction.

| Gene | Primer | Sequence(5'→3’) |
| --- | --- | --- |
| *GRP78* | Forward | ACCCATGCAGTTGTGACTGT |
|  | Reverse | ACATCGAAGGTTCCACCACC |
| *GAPDH* | Forward | AGACAGCCGCATCTTCTTGT |
|  | Reverse | TACGGCCAAATCCGTTCACA |
| *IRE1* | Forward | CGCAGGTGCAATGACATACAA |
|  | Reverse | GTCCACTTGATGGAGCCTGT |
| *XBP1s* | Forward | ACCAGGAGTTAAGGACACGC |
|  | Reverse | ACGTAGTCTGAGTGCTGCG |
| *ATF6* | Forward | CTCATGGACCAGGTGAAGACT |
|  | Reverse | ATGTCTGACTCCCAAGGCATC |
| *JNK* | Forward | AGGAGGTCCCAAAGCCTACT |
|  | Reverse | ACTATGCCTGCTCTGCTCAC |
| *CHOP* | Forward | GCAGCGACAGAGCCAAAATAA |
|  | Reverse | CTGCTTTCAGGTGTGGTGGT |
| *BCL-2* | Forward | CTTTGAGTTCGGTGGGGTCA |
|  | Reverse | CATCCCAGCCTCCGTTATCC |
| *Bax* | Forward | CGTCTGCGGGGAGTCAC |
|  | Reverse | ATCTGTTCAGAGCTGGTGGG |
| *Caspase-3* | Forward | GCGTAAGGAAAGGAGAGGTG |
|  | Reverse | ACAGACCAGTGCTCACAAGG |
| *Caspase-9* | Forward | GGCCTTCACTTCCTCTCAAG |
|  | Reverse | GGACACAAGGATGTCACTGG |
| *Caspase-12* | Forward | TGCAGAGGCAGACATACTGG |
|  | Reverse | CTGTCTCCACATGGGCCTTT |
| *NF-κB* | Forward | GGACAGCACCACCTACGATG |
|  | Reverse | CTGGATCACTTCAATGGCCTC |
| *TNFα* | Forward | CATGGATCTCAAAGACAACCAA |
|  | Reverse | CTCCTGGTATGAAATGGCAAAT |
| *IL6* | Forward | AAGGACCAAGACCATCCAAC |
|  | Reverse | ACCACAGTGAGGAATGTCCA |
| *ZO-1* | Forward | TGCCACACTGTGACCCTAAA |
|  | Reverse | GGACAGAAACACAGTTGGCT |
| *ZO-2* | Forward | TCCAAGAAGCACAGAATGCG |
|  | Reverse | CTCTGGGGGTCTCATGTGC |
| *Claudin-1* | Forward | GGACAACATCGTGACTGCTC |
|  | Reverse | CCAGCAGGATGCCAATTACC |
